# Prediction of Cover–Subsidence Sinkhole Volume Using Fibre Bragg Grating Strain Sensor Data

**DOI:** 10.3390/s25072272

**Published:** 2025-04-03

**Authors:** Wesley B. Richardson, Suné von Solms, Johan Meyer, Charis Harley

**Affiliations:** Department of Electrical and Electronic Engineering Science, University of Johannesburg, Johannesburg 2006, South Africa; svonsolms@uj.ac.za (S.v.S.); johanm@uj.ac.za (J.M.); charley@uj.ac.za (C.H.)

**Keywords:** cover–subsidence sinkhole, exploratory data analysis, extreme gradient boost, fibre bragg grating, machine learning, strain data, volume regression, XGBoost

## Abstract

Sinkholes are geohazards that commonly form in karstifiable terrain and are an ever-present danger to infrastructure and human life. This paper aims to answer the question: Can a cover–subsidence sinkhole’s volume be determined using fibre Bragg grating sensor strain data and machine-learning techniques? Exploratory data analysis was conducted on fibre Bragg grating sensor strain data collected from an experimental test rig whereby a cover–subsidence sinkhole was formed. It was found that statistical techniques and machine-learning algorithms that assume normality are inappropriate when performing phase classification and volume regression tasks on the cover–subsidence sinkhole when given fibre Bragg grating sensor’s strain data. Weighted Least Squares regression, Support Vector Regression, and eXtreme Gradient Boosting were implemented on the data during phase two of the cover–subsidence sinkhole formation to determine the volume of the sinkhole. Weighted Least Squares regression obtained the lowest $R^{2}$ values for training and testing. Support Vector Regression had significantly improved results over Weighted Least Squares regression, while eXtreme Gradient Boosting obtained the highest $R^{2}$ values for training and testing. The highest $R^{2}$ values for eXtreme Gradient Boosting obtained were 1.00 for training and 0.97 for testing. In addition, eXtreme Gradient Boosting had the lowest root mean squared errors compared to Weighted Least Squares regression and Support Vector Regression. It was found that eXtreme Gradient Boosting is a strong candidate for determining the volume of the C–S sinkhole when using fibre Bragg grating strain data.

## 1. Introduction

Sinkholes are common and naturally occurring geological hazards that are habitually responsible for the sudden and often devastating ground collapse and can potentially damage nearby infrastructure, cause loss of life, and threaten water and environmental resources [1,2,3]. Sinkholes commonly form in karstifiable rocks, such as carbonates (e.g., dolomite and limestone) and evaporites (e.g., salt, gypsum, and anhydrite) [1,4]. In the United States (US) alone, the annual cost due to damages caused by sinkholes exceeds $300 million [5]. However, the cost of damages caused by sinkholes in the US is expected to be higher due to the need for national tracking [5]. In the past fifty years in South Africa (SA), thousands of sinkholes, subsidence, and crack events have transpired in the Gauteng province (the country’s most populous province), whose surface area is approximately 23–24% dolomite [6,7]. According to Buttrick et al. [7], in 2011, a community of approximately 30,000 households was relocated to more stable ground (i.e., ground experiencing less instability due to dolomite), which exceeded the cost of US $600 million at that time. Buttrick et al. [7] further reported that approximately 4–5 million people worked and resided on dolomitic land in 2011 in SA.

The occurrence of sinkholes is worldwide [7], with 15.2% of the global ice-free continental surface containing karstifiable carbonate rock [4]. In 2020, Goldscheider et al. [4] reported that from a global perspective, 1.18 billion people (approximately 16.5% of the global population at that time) lived on karst terrain. Three common types of sinkholes form in karstifiable terrain: dissolution, cover–subsidence (C–S), and cover–collapse (C–C) [8]. It is also worth mentioning that a sinkhole can be a combination of the three common types of sinkholes [8].

Infrastructure that is highly susceptible to damage resulting from sinkhole formations is linear infrastructure (such as roads, bridges, and railways) [9,10,11]. In the US, roadways are especially susceptible to damage caused by sinkhole formations due to the presence of carbonate minerals and carbonate rock along the roadways [11]. According to Rizzo and Bryson [11], an essential aspect of preventing significant traffic caused by damage resulting from sinkholes is to have a system that detects soil raveling or voids early on. Regarding railways, the emergence of sinkholes along or under railway tracks may lead to high maintenance costs, service interruptions, and, worst case scenario, the derailment of the train, leading to loss of human life [10]. Guerrero et al. [10] list several factors that affect the probability of train derailment due to the formation of sinkholes occurring along the railway tracks. One of the factors Guerrero et al. [10] listed that affects the probability of train derailment is the vulnerability of the infrastructure (i.e., the railway tracks). The vulnerability of the infrastructure depends mainly on the incorporation and culmination of sinkhole protection measures in the design of the infrastructure, as well as the installation of sinkhole monitoring systems [10]. The sinkhole monitoring system, in this case, should be capable of detecting subtle ground deformations and anticipating sinkhole collapse [10].

As a result of the threat that sinkholes pose to infrastructure and lives, Sinkhole Detection and Monitoring Systems (SDMSs) must be implemented to ensure that engineering countermeasures can be planned and carried out to prevent catastrophic infrastructure damage and loss of life from occurring. The preceding statement is supported by the literature, where Möller et al. [9] state that monitoring systems that can provide a continuous record of potential indicators of sinkhole formation are recommended, and Nam et al. [12] state that early prediction and detection of sinkholes are pertinent measures for protecting citizens and infrastructure. In addition, Hoai et al. [13] state that accurate tracking of sinkholes is essential to protect human life and prevent property damage, and Möller et al. [9] further state that developing a sinkhole early warning detection system would protect infrastructure and save lives. Rizzo and Bryson [11] also state that early sinkhole detection along roadways will enable the Department of Transportation in the US to track the sinkhole’s growth rate, which will aid in deciding when to disrupt traffic flow for repairs to be conducted and budget for the repairs. Planning and budgeting the repairs enables a proactive maintenance approach instead of a reactive one.

Only the formation of the cover–subsidence (C–S) sinkhole is discussed in this paper as it is the type of sinkhole that is the paper’s focus. Understanding the formation process of the C–S sinkhole was essential for developing the experimental methodology, which the authors previously published in [1].

C–S sinkholes develop when carbonate bedrock (which contains openings or cavities) is covered in an overburden of permeable sediment and sand [8]. The granular sediment in the overburden spalls into the carbonate bedrock via the openings in the bedrock [8]. As the sediment continues to spall into the carbonate bedrock, a column of the sediment forms in cavities in the bedrock; this process is commonly referred to as *piping* [8]. As the process of infilling in the bedrock continues, a noticeable depression in the surface of the overburden forms [8]. The surface depression will grow as long as the infilling process continues, resulting in a surface depression that can be several meters in depth and diameter [8]. The resulting surface depression is a C–S sinkhole.

**Figure 1:**
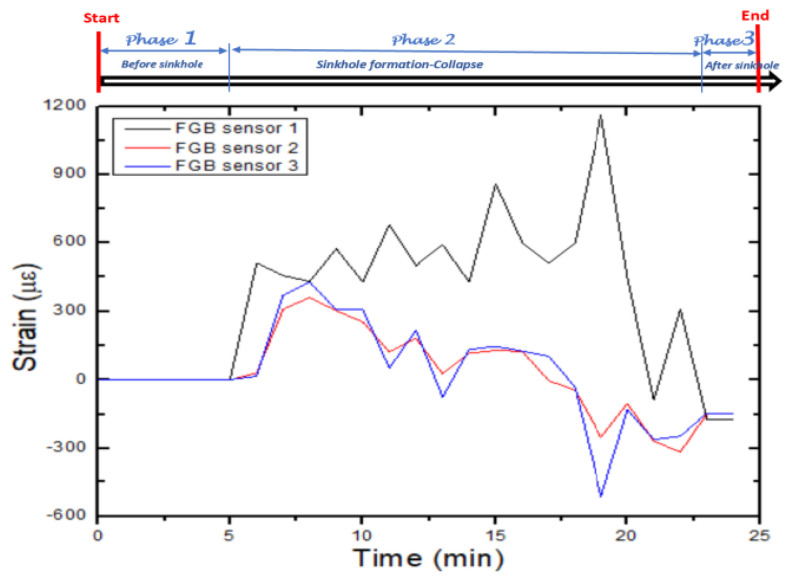
Induced strain during the CS sinkhole formation process. Source: [2].

Yumba et al. [2] published a conference paper in 2023 on the same experiment the authors did. Yumba et al. [2] utilized fibre Bragg grating (FBG) strain data gathered from a C–S sinkhole (sinkhole in dry sand) experiment and from another experiment where a cover–collapse sinkhole (sinkhole in wet sand) formed to compare the strain distribution of the two types of sinkholes (i.e., comparing the strain distribution of a cover–collapse sinkhole and a C–S sinkhole). According to Yumba et al. [2], the strain distribution along the vertical centerline above the C–S and cover–collapse sinkhole cavities provides insightful information into the deformation pattern of the soil. The strain values recorded by the FBG sensors, placed along the vertical centerline above the sinkhole cavity, indicated the extent and magnitude of the strain induced by the sinkhole event [2]. In other words, the strain recorded by the FBG sensors can help quantify the impact of the C–S sinkhole and provide insights into the physical changes happening in the surrounding area. Yumba et al. [2] found that strain variation for forming a C–S sinkhole had three phases: before, during, and after the sinkhole has formed. Figure 1 shows the induced strain during the three phases of the C–S sinkhole formation process.

Figure 1 shows that during phase one (before sinkhole formation), the strain recorded by the FBG sensors was constant and near zero; during phase two (during the sinkhole formation), the strain changed significantly [2]. During phase three (after C–S sinkhole formation has ceased), the strain values stabilized at different values.

The work presented in this paper contributes towards developing a sinkhole monitoring system that can geometrically characterize a sinkhole in terms of its volume using FBG sensor strain data. Of the three common types of sinkholes listed earlier, the current research study focuses on C–S sinkholes. The main aim of the current paper was to conduct an in-depth Exploratory Data Analysis (EDA) on all three phases of the collected FBG strain data and present a Machine-Learning (ML) model that can output the volume of the C–S sinkhole when given strain measurements from the FBG strain sensors. The purpose of conducting the more in-depth EDA was to develop an understanding of the connectivity, patterns, and relationships between the FBG strain data and the volume of the C–S sinkhole. From there, the developed understanding was used to guide our choice of a supervised ML algorithm to output the volume of the C–S sinkhole when given the FBG strain data.

The remainder of the paper is structured as follows: Section 2 discusses the experimental methodology used to collect the FBG strain and C–S sinkhole volume data from a C–S sinkhole, Section 3 discusses the theoretical background of the probabilistic and statistical analysis methods used to conduct an EDA on the collected FBG strain and C–S sinkhole volume data, Section 4 discusses the data analysis procedure, Section 5 discusses the data analysis results, Section 6 discusses the results from the Weighted Least Squares (LWS) regression, Support Vector Regression (SVR), and eXtreme Gradient Boosting (XGBoost) models which were fitted to the phase 2 data which modelled the volume of the C–S sinkhole, and Section 7 concludes the paper and discusses future work.

## 2. Materials and Methods of the C–S Sinkhole Formation

This section provides a comparative discussion with prior research studies, highlighting advancements introduced in the present work. In addition, this section discusses the experimental methodology used to collect the FBG sensor strain and C–S sinkhole volume data from a C–S sinkhole. Yumba et al. [14] conducted an experiment to investigate the FBG strain profile during a C–C sinkhole formation by placing 18 FBG strain sensors at varying levels above the cavity. Yumba et al. [14] placed nine FBG strain sensors vertically and nine FBG strain sensors horizontally above the cavity. It is important to note that the study conducted by Yumba et al. [14] focused on a C–C sinkhole formation rather than a C–S sinkhole formation. In addition, the study conducted by Yumba et al. [14] focused on investigating the strain profile during the C–C sinkhole formation rather than determining the volume of the C–C sinkhole. Möller et al. [9] conducted an experiment to investigate the feasibility of utilizing Distributed Fibre Optic Sensing (DFOS) as an early warning system for sinkholes. The type of sinkhole investigated by Möller et al. [9] was a C–S sinkhole because the setup utilized mimicked progressive subsurface migration of the overburden material into a void, which is characteristic of C–S sinkhole formations. Möller et al. [9] placed DFOS cables horizontally at various depths. It is important to note that the study conducted by Möller et al. [9] utilizes DFOS instead of FBG and that the DFOS cables were placed horizontally. In addition, the study conducted by Möller et al. [9] was not focused on using the data obtained from the DFOS cables to determine the volume of the C–S sinkhole but was instead focused on early warning detection. Labuschagne et al. [3] conducted a study investigating the use of FBG strain sensors for C–S sinkhole monitoring. While the study conducted by Labuschagne et al. [3] focused on a C–S sinkhole, the FBG strain sensors used in the study were placed horizontally above the cavity. Labuschagne et al. [3] used twelve FBG strain sensors across four fibre cables (3 FBG strain sensors per fibre cable). The fibre cables were placed horizontally at varying depths [3].

From the discussion above, the other studies involving optical fibre sensing and sinkholes were mainly focused on investigating strain profiles and the feasibility of using optical fibre sensing technology to detect sinkhole formations. None of the studies discussed above were focused on using the data collected from the sensors to determine the volume of a sinkhole. In addition, not all of the studies were focused on C–S sinkholes, and the studies that were focused on C–S sinkholes had placed the FBG strain sensors horizontally above the cavity and were only focused on investigating strain profiles. The novelty of the work presented in this paper is the orientation in which the FBG strain sensors were placed above the cavity and the approach of using the data collected from the FBG strain sensors to determine the volume of a C–S sinkhole. The FBG strain sensors were placed vertically above the cavity of the C–S sinkhole. The decision was made to install FBG strain sensors vertically rather than horizontally in order to minimize soil disturbance during installation. In field applications, horizontal installation would require removing large amounts of the surface layer, which increases the risk of triggering a sinkhole formation. Vertical placement reduces excavation effort and preserves ground stability. Furthermore, the work presented in this paper uses fewer FBG strain sensors than the studies discussed above. The study presented by Yumba et al. [14] used nine FBG strain sensors placed vertically above a C–C sinkhole cavity, and the study presented by Labuschagne et al. [3] used twelve sensors placed horizontally above a C–S sinkhole cavity. The work presented in this paper uses three FBG strain sensors on one fibre cable installed vertically above a C–S sinkhole cavity.

A C–S sinkhole was formed under controlled conditions in a laboratory by placing Cullinan Silica sand (obtained from Cullinan Mine, Pretoria, Gauteng, South Africa) over a water-filled balloon (which acted as the cavity) in a Perspex box, embedding FBG strain sensors vertically above the cavity, and allowing the water-filled balloon to deflate using a calibrated flow-valve. As the water-filled balloon deflated, FBG strain sensor measurements were recorded, and the volume of the C–S sinkhole was calculated. The deflation of the water-filled balloon allowed for a surface deformation to occur (i.e., a C–S sinkhole). More details on the experimental methodology can be found in [1]. It should be noted for the rest of the paper that FBG strains S1, S2, and S3 refer to FBG strain sensors 1, 2, and 3 from [1], respectively.

## 3. Theoretical Basis of Analytical Methods: Probability and Statistics

This section discusses the theoretical background of the probabilistic, statistical analysis methods and machine-learning algorithm(s) implemented on the data. To better understand the connectivity, patterns, and relationships between the FBG strain data and the C–S sinkhole volume, this section discusses the following analytical methods: Spearman’s Rank correlation coefficient, Kolmogorov–Smirnov (K–S) test, Shapiro–Wilk (S–W) test, and Anderson–Darling (A–D) test. The Kolmogorov–Smirnov, Shapiro–Wilk, and Anderson–Darling tests were used because they are the best-known and most common analytical tests to check for the normal distribution of data [15]. In this paper, the data were tested for normal distribution because, according to Jesussek and Volk-Jesussek [15], one of the most common assumptions for statistical tests is that the data utilized are normally distributed. The justification and objective for implementing each method are discussed, followed by an explanation of the respective method. The following section discusses Spearman’s Rank correlation coefficient.

### 3.1. Spearman’s Rank Correlation Coefficient

The reason for conducting correlation analysis (such as Spearman’s Rank) on the variables in the data is that if two variables show correlation, then tests can be carried out to determine if one of the variables can be used to predict the other variable by using regression [15]. In the case of this paper, correlation analysis helped determine if one or more of the FBG strain sensor measurements could determine the volume of the C–S sinkhole.

Spearman’s Rank correlation coefficient (also known as Spearman’s Rank or Spearman’s rho) is a nonparametric correlation analysis that determines the strength of the monotonic relationship between the ranked values of two variables [15,16,17,18]. Spearman’s rank is the nonparametric equivalent of Pearson’s correlation and is, thus, used when the assumptions necessary for Pearson’s correlation analysis are not met [15]. Spearman’s rank correlation coefficient, ρ, is calculated as follows [16,17,18]:

(1) $$\rho =1-\frac{6\sum_{i=1}^{n}d_{i}^{2}}{n\left(n^{2}-1\right)}$$

In $\left(1\right)$, n is the number of paired data points. $d_{i}$ is the difference between the ranks for each of the n pairs and is calculated as $d_{i}=R_{X_{i}}-R_{Y_{i}}$, where $R_{X_{i}}$ is the rank of $X_{i}$ and $R_{Y_{i}}$ is the rank of $Y_{i}\;$ [16,18]. The range of ρ is [−1,1] where if ρ<0, there is a negative monotonic relationship between the two variables, if ρ>0, there is a positive monotonic relationship between the two variables, and if ρ=0, there is no monotonic relationship between the two variables [15,16]. Table 1 may be used as a guide to aid in determining the strength of the correlation between two variables when using Spearman’s Rank correlation.

The following section introduces, defines, and discusses the K–S test.

### 3.2. The K–S Test

In this paper, the objective of carrying out the K–S test is to determine if the data collected from the FBG strain sensors and the C–S sinkhole volume data follow the normal distribution. Knowing what distributions the data follow (if any) will influence which statistical techniques and ML algorithms are appropriate for the collected data. For example, normally distributed data are a Pearson’s correlation coefficient analysis criterion for hypothesis testing. The K–S test can determine if the collected data follow a normal distribution [15]. The K–S test can also determine if the collected data follow other distributions, not just the normal distribution [15].

The K–S test is a nonparametric goodness-of-fit test that is used to determine if data from two samples come from the same unspecified distribution or if the distribution comes from a specific hypothesized (i.e., theoretical) distribution [17,18,20]. An example of the specific hypothesized distribution is the normal distribution. The only assumption of the K–S test is that the two distributions being compared are continuous [20]. Determining if two independent samples come from the same distribution using the K–S test is known as the two-sample K–S test, and determining if the distribution of a single sample follows a specific hypothesized distribution is known as the one-sample K–S test [21,22]. The basis for the K–S test is to determine the absolute maximum difference between the Cumulative Distribution Functions (CDFs) of the two samples being compared [17]. In the case of the one-sample method, we are comparing our sample data’s Empirical Distribution Function (EDF) against the CDF of the specific hypothesized distribution.

Consider the sample data $\left(x_{1},x_{2},\cdots ,x_{n}\right)$ of size n, whose unknown distribution function is denoted F(x), and whose EDF is denoted by H(x) [18]. The CDF of the specific hypothesized distribution is denoted by $F_{O}(x)$ [18]. The hypotheses for the two-sided K–S test are as follows [18]:

(2) $$H_{0}:F(x)=F_{O}(x)\;\forall \;x\;H_{a}:F(x)\neq F_{O}(x)\;for\;at\;least\;one\;x$$

In $\left(2\right)$, the null hypothesis, $H_{O}$, states there is no difference between F(x) and $F_{O}(x)$ [18]. In other words, the unknown distribution function of the sample data follows the specific and known hypothesized distribution (such as the normal distribution). The alternative hypothesis, $H_{a}$, states that the converse is true. The statistical test for the two-sided K–S test is defined as follows [18]:

(3) $$T=\underset{x}{\sup}\left|F_{O}(x)-H(x)\right|$$

In $\left(3\right)$, the value of T is applied to the decision rule of the two-sided K–S test. The decision rule for the K–S test is to reject $H_{O}$ if the following is true [18]:

(4) $$T>t_{n,1-\alpha }$$

In $\left(4\right)$, α is the significance level, t is a value that is read from the K–S table with parameters n and 1−α [18]. The K–S table can be found in [23]. If we fail to reject $H_{O}$, then F(x) is taken as being the same as $F_{O}(x)$. In other words, the distribution of the sample data follows the specific hypothesized distribution. The following section defines and discusses the S–W test.

### 3.3. The S–W Test

The reason for using the S–W test in conjunction with the K–S test is to provide added assurance on any conclusions made regarding the normality of the collected data. The S–W test is another common analytical method to check if data are normally distributed [15,17]. For the S–W test, the null and alternative hypothesis is the same as in $\left(2\right)$, where F(x) is the CDF of the collected data and $F_{O}(x)$ is the CDF of the normal distribution. In other words, the null hypothesis states that the data are normally distributed, and the alternative hypothesis states that the data are not normally distributed [15,24]. The test statistic for the S–W test is calculated as follows [25]:

(5) $$W=\frac{{\left(\sum_{i=1}^{n}a_{i}x_{\left(i\right)}\right)}^{2}}{\sum_{i=1}^{n}{\left(x_{\left(i\right)}-\bar{x}\right)}^{2}}$$

In $\left(5\right)$, $x_{\left(1\right)}\leq x_{\left(2\right)}\leq \cdots \leq x_{\left(n\right)}$ are the ordered observations of the data in increasing order, n is the size of the data, and $a_{i}$ is the tabulated coefficients [24,26]. The coefficients, $a_{i}$, for 2≤50 and 1≤i≤25 can be found in Table 5 in [25]. The test statistic, W, is then compared with critical values for W [24]. In this paper, we denote the critical value for W as $W_{crit}$. If $W>W_{crit}$, then we fail to reject the null hypothesis at a significance level, α [24]. The values for $W_{crit}$, for 3≤n≤50 can be found in Table 6 in [25]. The following section discusses the A–D test.

### 3.4. The A–D Test

The reason for doing the A–D test in conjunction with the K–S and S–W tests is to conduct a more comprehensive and exhaustive assessment of the normality of the data. Combining the results of the K–S test, S–W test, and A–D test will help confirm any conclusions on the data about normality.

Much like the K–S test, the A–D test is a goodness-of-fit test that determines if the EDF of the data follows a specific hypothesized theoretical distribution (such as the normal distribution) [15,17,18]. In order to test for the normal distribution using the A–D test, the data observations are first ordered in ascending order, after which the A–D test statistic is calculated [18]. The test statistic for the A–D test is calculated as follows [17,18]:

(6) $$A_{n}^{2}=-\frac{1}{n}\left[\sum_{i=1}^{n}\left(2i-1\right)\left\{\ln{(z}_{i})+\ln\left(1-z_{n+1-1}\right)\right\}\right]-n$$

In $\left(6\right)$, $A_{n}^{2}$ is the A–D test statistic, n is the sample size, and $z_{i}$ is the transformed ordered observation using the standard normal CDF, which is given by the following [17]:

(7) $$z_{i}=\Phi \left(\frac{x_{\left(i\right)}-\bar{x}}{s}\right)$$

In $\left(7\right)$, s is the sample standard deviation, and Φ(x) is the standard normal CDF that is defined as follows [17,27]:

(8) $$\Phi \left(x\right)=\int_{-\infty }^{x}\frac{1}{\sqrt{2\pi }}e^{-\frac{1}{2}u^{2}}du$$

The calculated A–D test statistic, $A_{n}^{2}$, is compared to the critical values from the A–D table for varying significance levels [18]. If $A_{n}^{2}$ is less than the critical value, we fail to reject the null hypothesis and conclude that it is highly unlikely that the data follow the normal distribution due to chance, and if $A_{n}^{2}$ is greater than the critical value, we reject the null hypothesis [18]. The following section discusses the Weighted Least Squares regression (WLS) algorithm, which was implemented on the collected data from the FBG strain sensors.

### 3.5. Weighted Least Squares Regression (WLS)

WLS is an extension of Ordinary Least Square (OLS) regression and is a parametric ML algorithm that accounts for uncertainty in observations by assigning weights to each data point [28,29]. Observations that possess high variance will be assigned a lower weight, while observations with lower variance (i.e., more reliable observations) will be assigned a higher weight [28]. Consequently, observations with higher variance have a reduced influence on the final coefficient estimations of the WLS model, whereas those with lower variance exert a greater influence [28]. The following section discusses the SVR algorithm, which was implemented on the collected data from the FBG strain sensors.

### 3.6. Support Vector Regression (SVR)

SVR is a supervised and generally considered nonparametric ML algorithm used to predict a numerical variable’s continuous value [30]. SVR utilizes the same principles as that of Support Vector Machines (SVM), and its objectives are to minimize prediction error and the value of the coefficients to prevent overfitting [30]. Essentially, the goal of the SVR ML algorithm is to find a suitable decision line (or hyperplane/hypersurface for data greater than two dimensions) that approximates the continuous-valued output of the target variable within a specified margin while simultaneously minimizing the model complexity. The following section discusses the XGBoost algorithm, which was implemented on the collected data from the FBG strain sensors.

### 3.7. eXtreme Gradient Boosting (XGBoost)

XGBoost is a scalable supervised ML algorithm and an ensemble tree model Gradient Boosting Machine (GBM) used in regression and classification tasks [31,32,33]. XGBoost utilizes boosting to learn from the errors (i.e., residuals) committed in previous decision trees [34]. In other words, XGBoost trains the next decision tree on the residuals from previous decision trees. The result is that multiple weak learners are combined to form a strong learner [31]. XGBoost is widely used by data scientists because it is currently one of the best gradient-boosted tree implementations available [31,32]. The following section discusses the data analysis procedure that was utilized in this paper.

## 4. Data Analysis Procedure

**Figure 2:**
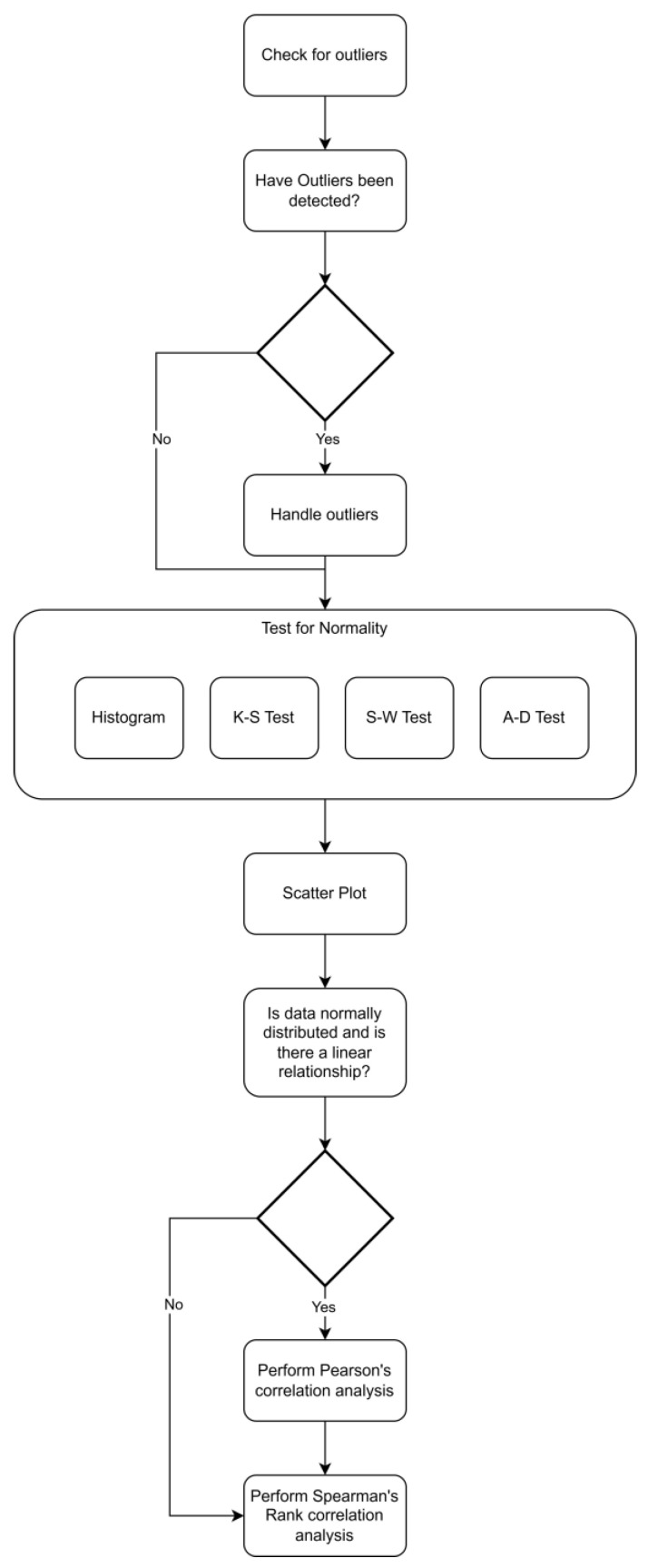
Data Analysis Procedure for C–S Sinkhole Formation Phases.

It should be noted that in the following discussions, the term “data” refers to the collected FBG strain measurements and the C–S sinkhole volume. The data were analyzed to determine the relationships amongst the variables (e.g., check if there was a correlation between the strain measurements from the different sensors and the C–S sinkhole volume) and to determine if the data were normally distributed. Collected data from the individual phases were analyzed in isolation (i.e., data from phase one were analyzed first, followed by phase two, and, lastly, phase three). After all the data from all the phases were analyzed in isolation, the data analysis results from all three phases were compared. The reason for comparing the data analysis results from all three phases is because it is desirable to understand how the forming C–S sinkhole may affect the data points over time (i.e., how the structure and relationships amongst the collected data change). Insights into how the C–S sinkhole formation affects the structural changes and patterns in the strain data aided in determining which ML algorithms are appropriate for determining the C–S sinkhole volume. Figure 2 shows the data analysis procedure conducted on the data in all three phases of the C–S sinkhole formation process.

As seen in Figure 2, the data analysis process began with outlier detection and handling of the outliers (i.e., deciding whether to remove the detected outliers or not). Outlier detection was achieved by using box plots. After conducting outlier detection, tests for normality were conducted. As can also be seen in Figure 2, the normality tests included analyzing the collected data’s histograms. The reason for including histograms as part of the data analysis procedure is that, according to Jesussek and Volk-Jesussek [15], analytical methods used to test for normal distribution (such as the K–S, S–W, and A–D tests) have significant drawbacks, and it is recommended to use analytical methods in conjunction with graphical methods (such as histograms and Quantile–Quantile plots). The collected data were tested for normality, and the relationships among the FBG strain sensors were also assessed. If the data were found to be linearly related and normally distributed, Pearson’s correlation coefficient was conducted to determine the strength of the correlation. After this, Spearman’s Rank correlation was conducted to determine the strength of the monotonic correlation between the FBG strain sensors and C–S sinkhole volume. DataTab [35] is an online subscription-based statistic calculator that was used to create the relevant visualizations (histograms and scatter plots) and perform the appropriate statistical test (K–S, S–W, and A–D tests as well as Pearson’s correlation coefficient and Spearman’s Rank correlation coefficient). DataTab was also utilized to conduct hypothesis testing on the calculated Pearson’s correlation and Spearman’s Rank correlation coefficients. Computational R was used to plot line charts. 

The following section presents and discusses the results obtained from the data analysis procedure.

## 5. Data Analysis Results and Discussion

**Figure 3:**
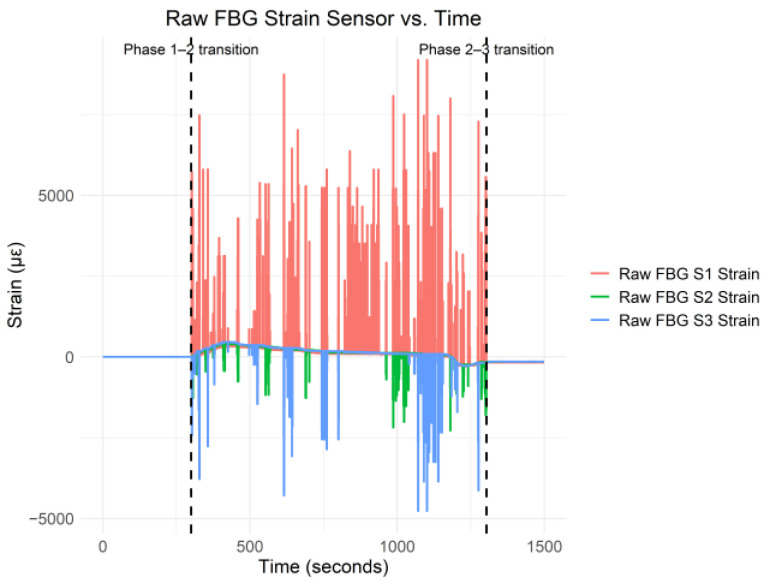
Raw FBG Strain S1–S3 Data vs. Time.

This section presents and discusses the data analysis results on all three phases of the C–S sinkhole formation process. Figure 3 shows the raw FBG strain data for FBG strain sensors 1–3 versus time.

**Figure 4:**
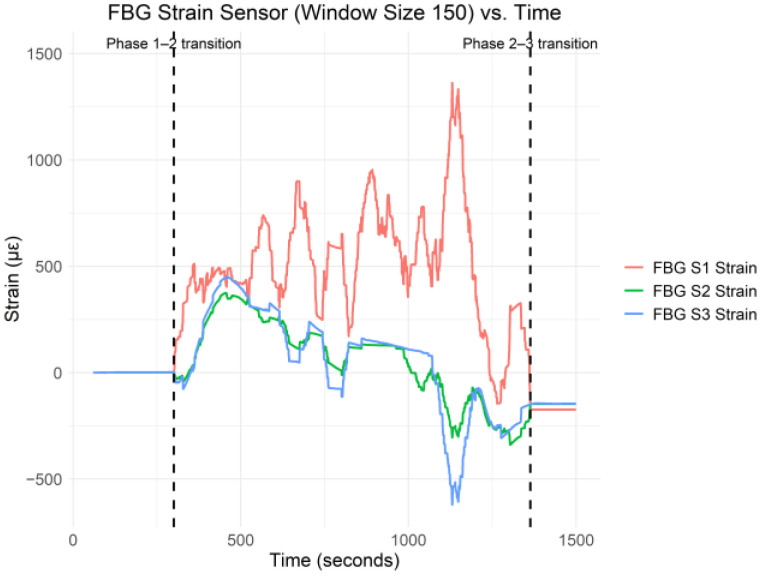
FBG Strain S1–S3 (Window Size 150) vs. Time.

In Figure 3, the three vertical dashed lines indicate the transition from phase one to two and phase two to three. As shown in Figure 3, there was significant strain during phase two of the C–S sinkhole formation. In [1], the authors reported that the C–S sinkhole formed for approximately 990 s. However, in Figure 3, phase two stopped 1003.6 s after the C–S sinkhole had started forming. Thus, there was a period of 13.6 s after the control valve had closed, and strain fluctuations were still recorded. The strain fluctuations during the 13.6 s period after the control valve was closed were due to the Cullinan Silica sand still settling. In order to have a clearer view of the strain data patterns and trends, a moving average with a window size of 150 was applied to the collected data. Figure 4 shows the moving average of the FBG strain data for sensors 1–3 with a window size of 150 versus time.

**Figure 5:**
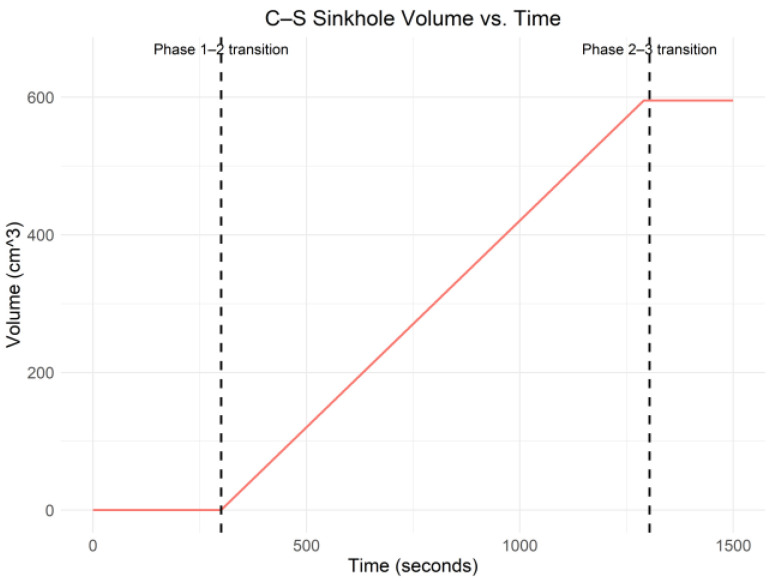
C–S Sinkhole Volume vs. Time.

Figure 4 shows that the FBG strain for all three sensors was relatively stable around the *x*-axis during phase one. During phase two, the FBG strain changed drastically, which was also found by Yumba et al. [2] when conducting a temporal analysis on the same dataset. In addition, during phase three, the strain for all three FBG strain sensors stabilized below the *x*-axis. Figure 5 shows the raw C–S sinkhole volume versus time.

In Figure 5, the volume of the C–S sinkhole was calculated using the methodology outlined in [1]. The volume of the C–S sinkhole is the inverse of the volume of the balloon (i.e., cavity). Figure 5 shows that the change in C–S sinkhole volume was linear, which was expected since the methodology used in [1] for calculating the volume was a linear function. The data collected during phases one, two, and three is represented by $S_{P1}$, $S_{P2}$, and $S_{P3}$, respectively. The dataset consists of 3750 data points. Each data point consists of the C–S sinkhole volume at that particular point during the experiment and the FBG strain measurements from $S_{P1}$, $S_{P2}$, and $S_{P3}$. Therefore, each data point has four features. In the dataset, we have the following: $\left|S_{P1}\right|=751$, $\left|S_{P2}\right|=2509$, and $\left|S_{P3}\right|=490$. Notably, $\left|S_{P2}\right|\gg \left|S_{P1}\right|\;$ and $\left|S_{P2}\right|\gg \left|S_{P3}\right|$. Therefore, when choosing ML algorithms to classify the data points as belonging to $S_{P1}$, $S_{P2}$, or $S_{P3}$, special consideration will have to be given to ML algorithms that are robust against imbalanced datasets. The following section presents and discusses outlier detection in the FBG strain data across all three phases of the C–S sinkhole formation process.

### 5.1. Outlier Detection in FBG Strain Data

Depending on the phase in which we are considering the strain data, S1, S2, or S3, we may observe either no notable outliers or some outliers above the upper whisker and/or below the lower whisker of a box plot. The decision was made across the board not to remove the outliers because: 1. The dataset is already imbalanced, and it is decided not to further imbalance it; 2. Outliers will impact statistical and ML implementation results, which may aid in understanding how robust future implemented ML models are to outliers in FBG strain data; and 3. The outliers may indicate new phenomena that warrant further investigation of the interaction and stress transfer mechanisms between fibre cables and soil during the various phases of the C–S sinkhole formation process. The following section presents and discusses the results of normality testing on the collected data during phase one.

### 5.2. Normality Test on FBG Strain Data During Phase One

**Figure 6:**
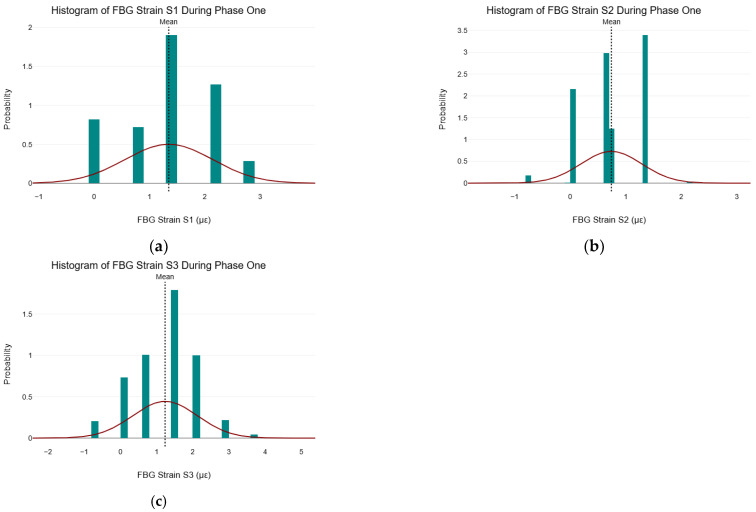
Histogram of FBG Strain measurements during Phase One. (**a**) Histogram of FBG strain S1 during phase one; (**b**) histogram of FBG strain S2 during phase one; (**c**) histogram of FBG strain S3 during phase one.

It should be noted that since this section analyzed data during phase one of the C–S sinkhole formation process (i.e., before the C–S sinkhole started forming), it was assumed that the C–S sinkhole volume was zero. As a result, no C–S sinkhole volume data were analyzed and presented during the phase one data analysis process. The assumption that the C–S sinkhole volume during phase one was zero aided in avoiding the inclusion of non-relevant metrics. Furthermore, during the phase one data analysis process, the focus was primarily on precursor conditions leading to the C–S sinkhole formation (i.e., phase two). Figure 6 shows the histograms of the FBG strain measurements for S1, S2, and S3 during phase one of the C–S sinkhole formation process with a normal distribution overlay and the locations of their respective means.

It can be seen in Figure 6 that the strain data from FBG S1, S2, and S3 did not follow a normal distribution because none of the histograms were symmetrical about the mean and did not follow the typical bell-shaped curve (shown by the normal distribution overlay).

Table 2 shows the K–S, S–W, and A–D test results for FBG strain S1 during phase one of the C–S sinkhole formation process. It should be noted that with regard to the p-values in Table 2, Table 3 and Table 4, the null hypothesis was that the data followed a normal distribution, and the alternative hypothesis was that the data did not follow a normal distribution. The significance level that was selected was 0.05. For the remainder of the paper and with regards to normality tests, it should be noted that $p_{K-S},\;{\;p}_{S-W}$, and $p_{A-D}$ represent the p-values for the K–S, S–W, and A–D tests, respectively.

From Table 2, the K–S, S–W, and A–D test results indicated that the data distribution for FBG strain S1 during phase one was not normally distributed, $T\left(\left[751\right]\right)=0.21$, $p_{K-S}<0.001$, $W\left(\left[751\right]\right)=0.90$,$\;p_{S-W}<0.001$ and $A_{n}^{2}\left(\left[751\right]\right)=28.03,\;p_{A-D}<0.001$. Table 3 shows the K–S, S–W, and A–D test results for FBG strain S2 during phase one of the C–S sinkhole formation.

From Table 3, the K–S, S–W, and A–D test results indicated that the data distribution for FBG strain S2 during phase one was not normally distributed, $T\left(\left[751\right]\right)=0.22,\;p_{K-S}<0.001$, $W\left(\left[751\right]\right)=0.85,\;p_{S-W}<0.001$ and $A_{n}^{2}\left(\left[751\right]\right)=46.74,\;p_{A-D}<0.001$. Table 4 shows the K–S, S–W, and A–D test results for FBG strain S3 during phase one of the C–S sinkhole formation.

From Table 4, the K–S, S–W, and A–D test results indicated that the data distribution for FBG strain S3 during phase one was not normally distributed, $T\left(\left[751\right]\right)=0.21,\;p_{K-S}<0.001$, $W\left(\left[751\right]\right)=0.93,\;p_{S-W}<0.001$ and $A_{n}^{2}\left(\left[751\right]\right)=22.91,\;p_{A-D}<0.001$.

All tests for normality (histograms, K–S test, S–W test, and A–D test) indicate that the strain data from all three FBG strain sensors deviate significantly from the normal distribution during phase one of the C–S sinkhole formation. The implication thereof is that statistical methods and ML algorithms that assume the normality of data should not be used during phase one of the formation of the C–S sinkhole. The following section discusses the scatter plots from the data collected during phase one.

### 5.3. Scatter Plots for FBG Strain During Phase One

**Figure 7:**
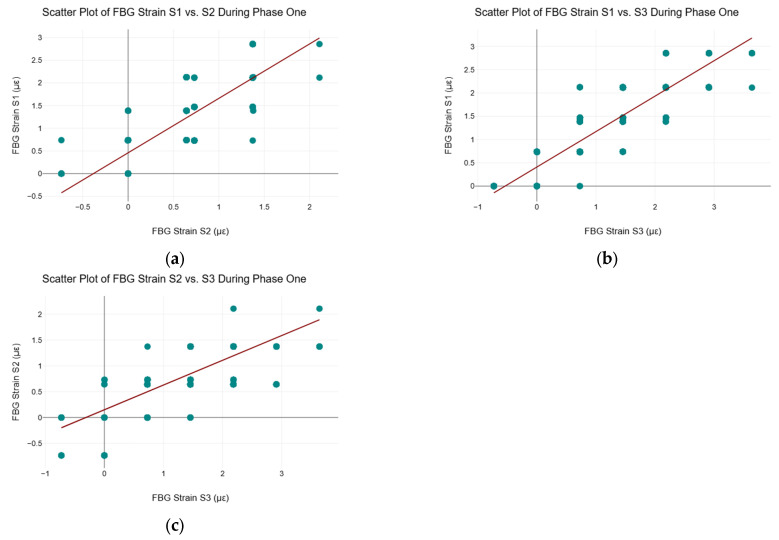
Scatter plots of FBG strain data during phase one. (**a**) Scatter plot of FBG strain S1 vs. S2 during phase one; (**b**) Scatter plot of FBG strain S1 vs. S3 during phase one; (**c**) Scatter plot of FBG strain S2 vs. S3 during phase one.

Figure 7 shows the scatter plots and regression lines between all three FBG strain sensors during phase one of the C–S sinkhole formation process.

Figure 7 shows that all three FBG strain sensors exhibited a high positive linear correlation with respect to each other during phase one of the C–S sinkhole formation process, which was expected since, as can be seen in Figure 3 and Figure 4, all three FBG strain sensors were approximately stable around the *x*-axis. The high positive linear correlation implies that linear ML regression algorithms may be appropriate for the data. However, high levels of multicollinearity may be present, given the high positive linear correlations between the sensors. To conduct hypothesis testing with Pearson’s correlation coefficient, the variables (i.e., the FBG strain data in this case) must be normally distributed, and there must be a linear relationship between the variables [15]. Thus, while Pearson’s correlation coefficient could be calculated, no statistical inferences from the correlation could be made due to the FBG strain data not following the normal distribution. As such, Pearson’s correlation coefficient analysis was not conducted on the FBG strain data collected during phase one of the C–S sinkhole formation process.

### 5.4. Spearman’s Rank Correlation Analysis During Phase One

Table 5 provides Spearman’s Rank correlation analysis results between the three FBG strain sensors and their corresponding *p*-values. For the calculated *p*-values, the null hypothesis was that there was no monotonic correlation between the FBG strain sensors, and the alternative hypothesis was that there was a monotonic correlation at a significance level of 0.05 between the FBG strain sensors during phase one.

Table 5 shows a very high positive monotonic correlation between all three FBG strain sensors during phase one. The correlation between all three FBG strain sensors was statistically significant, with all their p-values being less than 0.001. The statistically significant and very high positive correlations between the three FBG strain sensors indicate that high multicollinearity levels might be present in the data during phase one. In regression analysis, multicollinearity refers to the situation when two or more predictor variables are highly correlated (i.e., one predictor variable can be represented by a linear combination of the other predictor variables) [15,17,18,36]. Multicollinearity amongst predictor variables may result in regression coefficients being unstable and not interpretable, and in the case where the predictor variables’ relations are rigorously linear, the coefficients become biased [15,18]. It is pertinent to note that multicollinearity does not impact the prediction outcome in regression analysis [15]. The degree of multicollinearity we may be seeing here makes sense, given that the sensors were monitoring the same phenomenon, were placed vertically above the cavity, and were on the same fibre cable. Since the FBG strain sensors were on the same fibre cable, the FBG strain sensors were not independent. Hence, high levels of multicollinearity were expected.

### 5.5. Normality Test on FBG Strain Data During Phase Two

**Figure 8:**
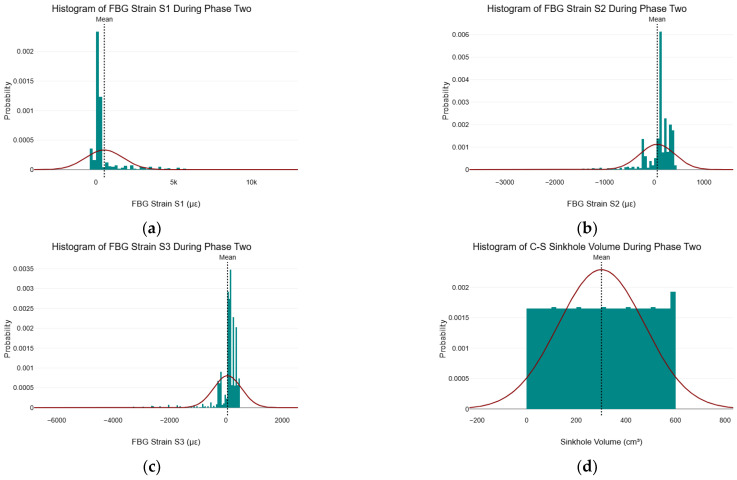
Histogram of FBG strain and C–S sinkhole volume during phase two. (**a**) histogram of FBG strain S1 during phase two; (**b**) histogram of FBG strain S2 during phase two; (**c**) histogram of FBG strain S3 during phase two; (**d**) histogram of C–S sinkhole volume during phase two.

Figure 8 shows the histograms of the FBG strain data for S1, S2, and S3, as well as the C–S sinkhole volume during phase two with a normal distribution overlay and locations of their respective means. Upon visual inspection, it was evident that the histograms in Figure 8a–c did not conform to the normal distribution overlay. Specifically, the histograms exhibited pronounced peaks around their respective means. Furthermore, the histograms were not symmetrical around their respective means. From (Figure 8d, it can be seen that while the histogram of the C–S sinkhole volume was approximately symmetrical about the mean, the histogram did not conform to the normal distribution overlay and exhibited a more uniform distribution.

Table 6 shows the K–S, S–W, and A–D test results for FBG strain S1 during phase two of the C–S sinkhole formation process. It should be noted that, with regard to the *p*-values in Table 6, Table 7, Table 8 and Table 9, the null hypothesis was that the data followed a normal distribution, and the alternative hypothesis was that the data did not follow a normal distribution.

From Table 6, the K–S and S–W test results indicate that the data distribution for FBG strain S1 during phase two was not normally distributed, $T\left(\left[2509\right]\right)=0.38,\;p_{K-S}<0.001$ and $W\left(\left[5209\right]\right)=0.53,\;p_{S-W}<0.001$. However, the A–D test result indicates that the data distribution for FBG strain S1 was normally distributed during phase two, $A_{n}^{2}\left(\left[2509\right]\right)=453.07,\;{\;p}_{A-D}=1$. Given that the K–S test and S–W test, along with visual inspection of the histogram, indicate that the data distribution for FBG strain S1 during phase two was not normally distributed, we reject the null hypothesis and state that it is highly unlikely that the alternative hypothesis is due to chance during phase two.

The contradictory normality test results between the K–S test, S–W, histogram, and A–D test highlight the importance of conducting more than one test for normality on the collected data, as they increase confidence in any conclusions made regarding the normality of the data. Table 7 shows the K–S, S–W, and A–D test results for FBG strain S2 during phase two of the C–S sinkhole formation process.

From Table 7, the K–S, S–W, and A–D test results indicated that the data distribution for FBG strain S2 during phase two was not normally distributed, $T\left(\left[2509\right]\right)=0.25$, $p_{K-S}<0.001$, $W\left(\left[2509\right]\right)=0.69$,$\;p_{S-W}<0.001$ and $A_{n}^{2}\left(\left[2509\right]\right)=217.07,\;p_{A-D}<0.001$. Table 8 shows the K–S, S–W, and A–D test results for FBG strain S3 during phase two of the C–S sinkhole formation.

From Table 8, the K–S and S–W test results indicate that the data distribution for FBG strain S3 during phase two was not normally distributed, $T\left(\left[2509\right]\right)=0.30,\;p_{K-S}<0.001$ and $W\left(\left[5209\right]\right)=0.30,\;p_{S-W}<0.001$. However, the A–D test result on DataTab returned infinity for the test statistic. Indicating that the test statistic had become excessively large, resulting in the impossible computation of the p-value. Given that three of the four tests (K–S test, S–W test, and histogram) indicate that the data distribution for FBG strain S3 during phase two was not normally distributed, we reject the null hypothesis and state that it is highly unlikely that the alternative hypothesis is due to chance during phase two. Table 9 shows the K–S, S–W, and A–D test results for the C–S sinkhole volume during phase two.

From Table 9, the K–S, S–W, and A–D test results indicate that the C–S sinkhole volume data distribution during phase two was not normally distributed, $T\left(\left[2509\right]\right)=0.06,\;{\;p}_{K-S}<0.001$, $W\left(\left[2509\right]\right)=0.95,\;{\;p}_{S-W}<0.001$ and $A_{n}^{2}\left(\left[2509\right]\right)=28.12,\;p_{A-D}<0.001$. We reject the null hypothesis for the C–S sinkhole volume and state that it is highly unlikely that the alternative hypothesis is due to chance during phase two.

Tests for normality indicated that the strain data from all three FBG strain sensors and the C–S sinkhole volume deviated significantly from the normal distribution during phase two of the C–S sinkhole formation. The implication thereof is that statistical methods and ML algorithms that assume the normality of data should not be used during phase two of the formation of the C–S sinkhole. The following section presents and discusses the scatter plots obtained from the data collected during phase two.

### 5.6. Scatter Plots of FBG Strain and C–S Sinkhole Volume During Phase Two

**Figure 9:**
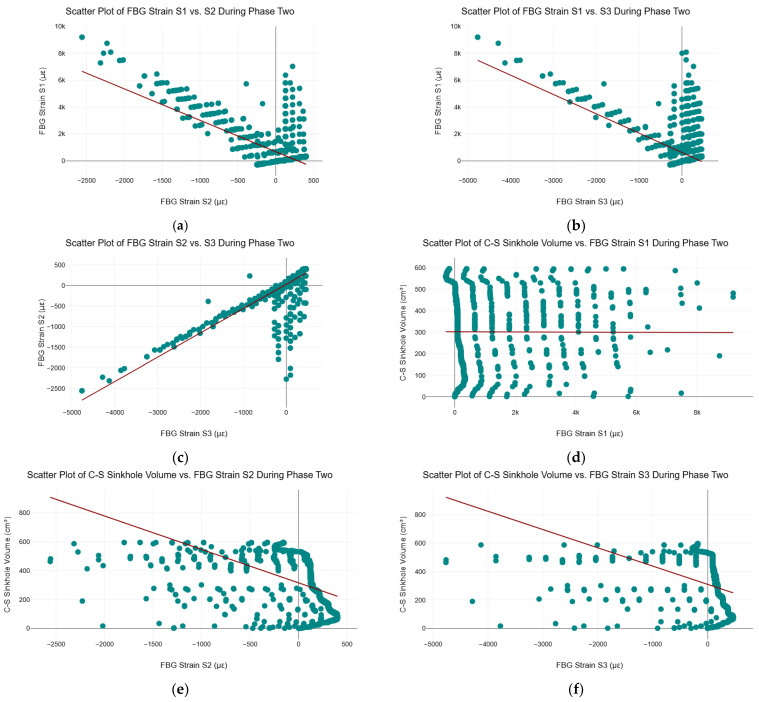
Scatter Plots of FBG Strain and C–S Sinkhole Volume during Phase Two. (**a**) Scatter plot of FBG strain S1 vs. S2 during phase two; (**b**) Scatter plot of FBG strain S1 vs. S3 during phase two; (**c**) Scatter plot of FBG strain S2 vs. S3 during phase two; (**d**) Scatter plot of FBG strain S1 vs. C–S sinkhole volume during phase one; (**e**) Scatter plot of FBG strain S2 vs. C–S sinkhole volume during phase two; and (**f**) Scatter plot of FBG strain S3 vs. C–S sinkhole volume during phase two.

Figure 9 shows the scatter plots and regression lines between all three FBG strain sensors and the C–S sinkhole volume during phase two of the C–S sinkhole formation process. Figure 9a–c indicate that the FBG strain sensors exhibited no strict linear correlation during phase two. The areas where FBG strain S1 exhibited a negative linear correlation with FBG strain S2 and S3 were expected because, as seen in Figure 4, the strain profile of FBG strain S1 is inversely proportional (i.e., mirrored) to the strain profiles of FBG strain S2 and S3. As FBG strain S1 experienced an increase in tensile strain, FBG strain S2 and S3 experienced an increase in compressive strain. The increase in compressive strain for FBG strain S2 and S3 was expected because, as the C–S sinkhole continued to grow in size, more and more of the Cullinan Silica sand was displaced closer towards the cavity, where FBG strain S2 and S3 were located. In other words, as the C–S sinkhole grew, FBG strain S2 and S3 were more compacted by the Cullinan Silica sand due to the piping process. FBG strain S1 experienced the opposite. FBG strain S1 was located closer to the surface; as the C–S sinkhole grew, FBG strain S1 was stretched due to the piping process. A resulting implication is that there is an inflection point between FBG strain S1 and S2 whereby the direction of the strain was reversed.

The regression line in Figure 9d indicates that there was no linear relationship between FBG strain S1 and the C–S sinkhole volume during phase two. In Figure 9e,f, although linear regression lines were fitted to the scatter plots, the data distribution did not exhibit a clear linear correlation between FBG strain S1 and S2 and the C–S sinkhole volume. The variability and dispersion of the data points indicate that a linear model does not adequately describe the relationship between these variables. Thus, linear ML algorithms are not appropriate for use during phase two of the C–S sinkhole formation to determine the volume of the C–S sinkhole. Similarly to phase one, the FBG strain data and C–S sinkhole volume data were found to be not normally distributed, resulting in Pearson’s correlation coefficient analysis not being conducted on the phase two data since no statistical inferences could be made from Pearson’s correlation coefficient.

### 5.7. Spearman’s Rank Correlation Analysis During Phase Three

Table 10 provides Spearman’s Rank correlation analysis results between the three FBG strain sensors and the C–S sinkhole volume and their corresponding p-values during phase two. For the calculated p-values, the null hypothesis was that there was no monotonic correlation between the FBG strain sensors, and the alternative hypothesis was that there was a monotonic correlation at a significance level of 0.05 between the FBG strain sensors during phase two.

From Table 10, Spearman’s Rank correlation showed a statistically significant and very high, positive monotonic correlation between FBG strain S3 and S2 during phase two. Spearman’s Rank correlation also showed that FBG strain S1 exhibited a statistically significant and moderate positive monotonic correlation between FBG strain S2 and S3. The results of Spearman’s Rank correlation analysis between all of the FBG strain sensors during phase two were found to be statistically significant, with all of their respective p-values being less than 0.001. Thus, we reject the null hypothesis and conclude that the alternative hypothesis (i.e., a monotonic correlation between the FBG strain sensors during phase two) is highly unlikely to be due to chance.

From Table 10, Spearman’s Rank correlation showed a statistically significant and negatively moderate correlation between FBG strain S1 and the C–S sinkhole volume during phase two. Spearman’s Rank correlation analysis also found that the C–S sinkhole volume exhibited a statistically significant and very high negative correlation between FBG strain S2 and S3. The results of Spearman’s Rank correlation analysis between all of the FBG strain sensors and the C–S sinkhole volume during phase two were found to be statistically significant, with all of their respective p-values being less than 0.001. Thus, we reject the null hypothesis and conclude that the alternative hypothesis (i.e., a monotonic correlation between the FBG strain sensors and the C–S sinkhole volume) is highly unlikely to be due to chance. Hence, we cannot definitively claim that the strain data cannot be utilized as predictors for the C–S sinkhole volume during phase two. Thus, nonparametric ML algorithms should be used when determining the C–S sinkhole volume, given the raw FBG strain data.

### 5.8. Normality Test on FBG Strain Data During Phase Three

**Figure 10:**
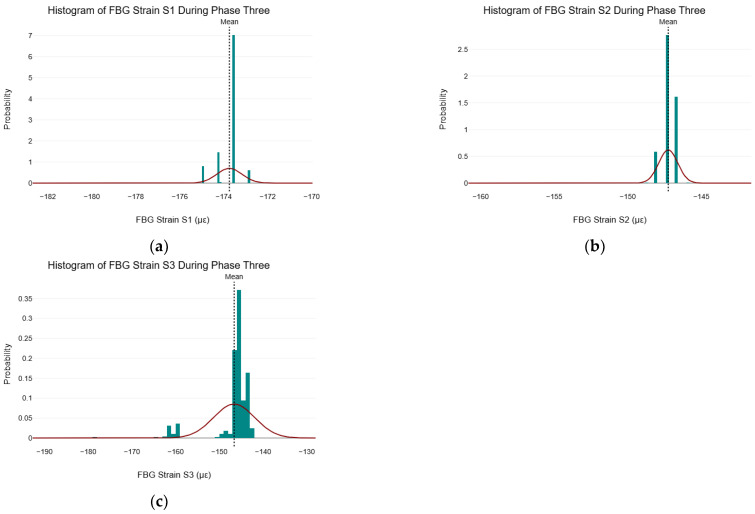
Histograms of FBG strain data during phase three. (**a**) Histogram of FBG strain S1 during phase three; (**b**) Histogram of FBG strain S2 during phase three; and (**c**) Histogram of FBG strain S3 during phase three.

In this section and the subsequent sections, FBG strain data collected during phase three of the C–S sinkhole formation process were analyzed by following the data analysis procedure outlined in Figure 2. During phase three, the formation of the C–S sinkhole ceased. As a result, the C–S sinkhole’s volume was constant and not analyzed further. Figure 10 shows the histograms of the FBG strain from S1, S2, and S3 during phase three of the C–S sinkhole formation process, with a normal distribution overlay and the locations of their respective means.

It can be seen in Figure 10 that the strain data from FBG S1, S2, and S3 did not follow a normal distribution due to asymmetry and skewness and because the histograms did not follow the typical bell-shaped curve (shown by the normal distribution overlay).

Table 11 shows the K–S, S–W, and A–D test results for FBG strain S1 during phase three of the C–S sinkhole formation process. It should be noted that with regard to the p-values in Table 11, Table 12 and Table 13, the null hypothesis was that the data followed a normal distribution, and the alternative hypothesis was that the data did not follow a normal distribution. The significance level that was selected was 0.05.

From Table 11, the K–S and S–W test results indicate that the data distribution for FBG strain S1 during phase three was not normally distributed, $T\left(\left[490\right]\right)=0.40,\;p_{K-S}<0.001$ and $W\left(\left[490\right]\right)=0.64,\;p_{S-W}<0.001$. However, the A–D test result on DataTab returned infinity for the test statistic. Indicating that the test statistic had become excessively large, resulting in the impossible computation of the p-value. Given that three of the four tests (K–S test, S–W test, and histogram) indicate that the data distribution for FBG strain S1 during phase three was not normally distributed, we reject the null hypothesis and state that it is highly unlikely that the alternative hypothesis is due to chance during phase three for FBG strain S1. Table 12 shows the K–S, S–W, and A–D test results for FBG strain S2 during phase three.

From Table 12, the K–S and S–W test results indicate that the data distribution for FBG strain S2 during phase three was not normally distributed, $T\left(\left[490\right]\right)=0.30,\;{\;p}_{K-S}<0.001$ and $W\left(\left[490\right]\right)=0.57,\;{\;p}_{S-W}<0.001$. However, similarly to FBG strain S1, the A–D test result on DataTab returned infinity for the test statistic. Given that three of the four tests (K–S test, S–W test, and histogram) indicate that the data distribution for FBG strain S2 during phase three was not normally distributed, we reject the null hypothesis and state that it is highly unlikely that the alternative hypothesis is due to chance during phase three for FBG strain S2. Table 13 shows the K–S, S–W, and A–D test results for FBG strain S3 during phase three.

From Table 13, the K–S, S–W, and A–D test results indicate that the data distribution for FBG strain S3 during phase three was not normally distributed, $T\left(\left[490\right]\right)=0.38,\;{\;p}_{K-S}<0.001$, $W\left(\left[490\right]\right)=0.5,\;{\;p}_{S-W}<0.001$ and $A_{n}^{2}\left(\left[490\right]\right)=87.74,\;{\;p}_{A-D}<0.001$. Thus, we reject the null hypothesis and state that it is highly unlikely that the alternative hypothesis is due to chance during phase three for FBG strain S3.

Tests for normality indicated that the strain data from all three FBG strain sensors deviated significantly from the normal distribution during phase three of the C–S sinkhole formation. The implication thereof is that statistical methods and ML algorithms that assume the normality of data should not be used during phase three of the formation of the C–S sinkhole. The following section discusses the scatter plots from the data collected during phase three.

### 5.9. Scatter Plots of FBG Strain Data During Phase Three

**Figure 11:**
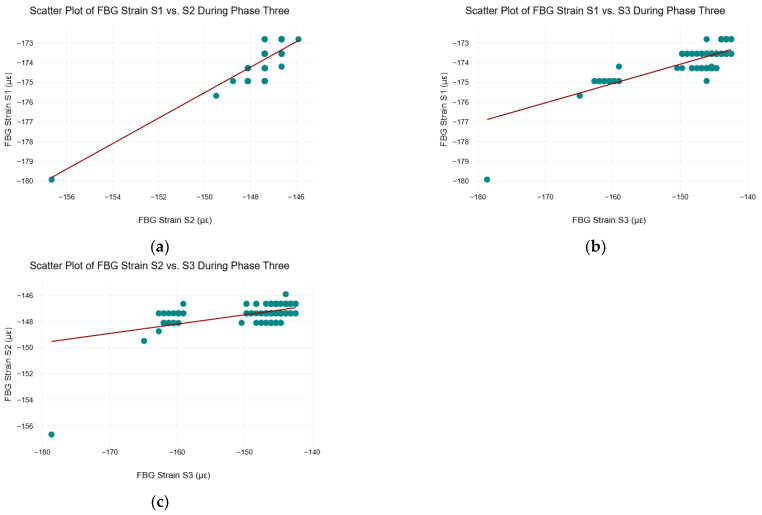
Scatter plots of FBG strain data during phase three. (**a**) Scatter plot of FBG strain S1 vs. S2 during phase three; (**b**) Scatter plot of FBG strain S1 vs. S3 during phase three; and (**c**) Scatter plot of FBG strain S2 vs. S3 during phase three.

Figure 11 shows the scatter plots of the FBG strain sensors during phase three of the C–S sinkhole formation process.

Figure 11a indicated that there was a strong positive linear correlation between FBG strain S1 and S2 during phase three. In Figure 11b,c indicated that FBG strain S3 exhibited a weak to moderate correlation with FBG strain S2 and S1, respectively. The correlations shown can be explained by the fact that the C–S sinkhole has stopped forming, and, as shown in Figure 3 and Figure 4, all three FBG strain sensors stabilized during phase three.

Similarly to phases one and two, the FBG strain data were found to be not normally distributed, resulting in Pearson’s correlation coefficient analysis not being performed on the phase three data since no statistical inferences could be made from Pearson’s correlation coefficient.

### 5.10. Spearman’s Rank Correlation During Phase Three

Table 14 provides Spearman’s Rank correlation analysis results between the three FBG strain sensors and their corresponding p-values during phase three. For the calculated p-values, the null hypothesis was that there was no monotonic correlation between the FBG strain sensors, and the alternative hypothesis was that there was a monotonic correlation at a significance level of 0.05 between the FBG strain sensors during phase three. From Table 14, FBG strain S1 exhibited a very high and monotonically positive correlation with FBG strain S3. Also, in Table 14, FBG strain S2 exhibited moderate and monotonically positive correlations with FBG strain S1 and S3.

All of the results from Spearman’s rank correlation analysis were statistically significant since all of the respective p-values between the FBG strain sensors were less than the significance level 0.05. Therefore, we reject the null hypothesis and conclude that it is highly unlikely that the alternative hypothesis is due to chance during phase three.

### 5.11. Phase One, Two, and Three Data Analysis Comparison

Across all three phases, it was found that none of the collected data followed the normal distribution. In phase one, the scatter plots indicated a positive linear correlation between all three FBG strain sensors. However, during phase two, the correlations did not remain the same, with none of the FBG strain sensors exhibiting a strict linear correlation with respect to each other. No strict linear relationship was found between the C–S sinkhole volume and the FBG strain sensors. During phase three, the three FBG strain sensors exhibited weak to moderate linear correlations with respect to each other. However, since the criteria for normality were not met in any of the phases, Pearson’s correlation analysis could not be conducted in any of the three phases. As such, statistical and ML techniques that assume normality and linearity should not be used on the collected data in any of the three phases. During phase one, all three FBG strain sensors exhibited statistically significant high to very high monotonically positive correlations with respect to each other. However, during phase two, the monotonic correlation between FBG strain S2 and S3 increased while the monotonic correlation between FBG strain S1 decreased with respect to FBG strain S2 and S3. The reason is that, during phase one, all three FBG strain sensors were stable around the *x*-axis. During phase two, FBG strain S1 experienced predominantly tensile strain. In contrast, FBG strain S2 and S3 experienced lower tensile strain levels and transitioned from tensile strain to compressive strain earlier than FBG strain S1. During phase three, all three FBG strain sensors experienced statistically significant moderate to very high monotonically positive correlations with respect to each other. The following section presents and discusses the results of ML implementation during phase two.

## 6. ML Implementation Results and Discussion

The EDA conducted in phase two led to the following two conclusions regarding the collected phase two data:

1. The strain data from all three FBG strain sensors and the C–S sinkhole volume deviate significantly from the normal distribution during phase two of the C–S sinkhole formation process.
2. The moderate to very high, monotonically negative correlation between the C–S sinkhole volume and all three FBG strain sensors is highly unlikely to be due to chance.

From these results, we have reason to believe we can determine the C–S sinkhole volume in phase two with the FBG strain data as predictors. Importantly, since the data are not normally distributed, we have to choose a model which does not rely on this assumption. The train Root Mean Squared Error (RMSE), test RMSE, and coefficient of determination ($R^{2}$) on the train, and test data were recorded for each ML algorithm implementation. The following section discusses the implementation results of the WLS algorithm on the collected data.

### 6.1. WLS Implementation Results

WLS was implemented on the collected phase two data to form a baseline to compare all implemented ML algorithm results. The WLS algorithm was implemented in computational R. All three FBG strain sensors were implemented as predictor variables, and the C–S sinkhole volume was the target variable. The weights were calculated by taking the inverse of the estimated error variance. The WLS algorithm was first implemented without a penalty term. In addition to implementing the WLS algorithm without a penalty term, regularization techniques were also implemented to prevent overfitting and enhance the WLS model’s ability to generalize to unseen data. The two regularization methods implemented were lasso regression (i.e., L1 regularization) and ridge regression (i.e., L2 regularization). The optimal regularization strength for L1 and L2 regularization was determined by performing 10-fold cross-validation. The results of the WLS implementation are shown in Table 15.

Table 15 shows that the WLS algorithm produced low $R^{2}$ values (all ≤ 0.45) for both training and testing. These results indicate that the WLS model failed to explain more than 50% of the variance in the collected data. Therefore, given the results of the WLS implementation, the current implementation of the WLS algorithm in this research study is not suitable for determining the volume of the C–S sinkhole when using FBG sensor strain data. Overall, the WLS model exhibited a poor fit, as empirically shown by the low $R^{2}$ and high RMSE values. The poor fit of the WLS model was expected because the EDA that was conducted revealed that the data exhibited a non-linear relationship during phase two of the C–S sinkhole formation. The following section discusses the results of the implementation of the SVR algorithm on the collected data.

### 6.2. SVR Implementation Results

As with the WLS algorithm, the SVR algorithm was implemented in computational R, all three FBG strain sensors were implemented as predictor variables, and the C–S sinkhole volume was the target variable. The Radial Basis Function (RBF) kernel was used when implementing the SVR algorithm. The RBF kernel was chosen because it is the most frequently used kernel when implementing the SVM and SVR algorithms [30]. The results of the SVR implementation are shown in Table 16.

In Table 16, ϵ controls the margin of tolerance where errors are ignored. Table 16 shows that the SVR algorithm overall outperformed the WLS algorithm based on the RMSE and $R^{2}$ values for both training and testing. The results of the SVR implementation indicate that the SVR algorithm is a potential candidate for determining the volume of a C–S sinkhole when using FBG strain data. However, further refinement is needed to improve $R^{2}$ values for both training and testing, ensuring the model accounts for more variance in the collected data. Further refinement would include investigating other kernels for implementation. Additionally, regarding determining the volume of the C–S sinkhole, more sophisticated methods could enhance accuracy and improve the model’s overall fit. The following section discusses the results of the implementation of the XGBoost algorithm on the collected data.

### 6.3. XGBoost Implementation Results

We decided to implement XGBoost due to the nonparametric nature of the decision trees used in its model. The XGBoost algorithm was implemented in computational R. All three FBG strain sensors were implemented as predictor variables, and the C–S sinkhole volume was the target variable. XGBoost was implemented using various training set sizes and learning rates. The maximum tree depth was set to 6, the maximum number of boosting rounds was set to 200, and the early stopping rounds were set to 10 (i.e., training stopped when there was no improvement in the RMSE for over 10 rounds). The results of the XGBoost implementation are shown in Table 17, with the most accurate implementation highlighted in bold.

From Table 17, it can be seen that extremely high $R^{2}$ values were obtained for all of the various XGBoost implementations. The best XGBoost implementation had a training set size of 80% and a test set size of 20% of the data collected during phase two, a learning rate of 0.3, the lowest train RMSE (7.20), third lowest test RMSE (30.74) as well as the one of the highest train and test $R^{2}$ values of 1.00 and 0.97, respectively. The test $R^{2}$ value of 0.97 indicates that 97%  of the variance in the C–S sinkhole volume can be explained by the XGBoost model when all three FBG strain sensors are used as predictor variables. Thus, XGBoost effectively captures the relationship between the C–S sinkhole volume and all three FBG strain sensors. Overall, XGBoost outperformed WLS and SVR due to the low RMSE and high $R^{2}$ values it obtained for training and testing. It was expected that XGBoost would outperform WLS and SVR as it is renowned for its accuracy and efficiency, which results from its optimization and regularization techniques. In addition, XGBoost also implements regularization to prevent the model from overfitting and, thus, improve model accuracy [37]. XGBoost’s efficiency stems from its parallel processing capabilities, which allow for parallel tree construction [33].

## 7. Conclusions and Future Work

The data collected across all three phases were not normally distributed, and the FBG strain sensors did not maintain strict linear correlations with respect to each other. Therefore, statistical techniques and parametric ML algorithms, which require the data to be normally distributed and the predictor variables to be linearly correlated, are inappropriate for the collected data. The FBG strain sensors exhibited statistically significant and moderate to very high monotonic correlations with respect to each other across all three phases. High levels of multicollinearity may be present due to statistically significant and very high monotonic correlations between the three FBG strain sensors across the different phases. All of the FBG strain sensors exhibited statistically significant and medium to very high monotonic correlations with respect to the C–S sinkhole volume during phase two. Therefore, we cannot definitively claim that the strain data cannot be utilized as predictors for the C–S sinkhole volume during phase two. WLS, SVR, and XGBoost were fitted to phase two of the collected data. WLS obtained the lowest $R^{2}$ values and the highest RMSE values. SVR showed significant improvement over WLS. XGBoost effectively captured the relationship between the FBG strain sensors and the C–S sinkhole volume. Thus, XGBoost is a strong candidate for determining the volume of the C–S sinkhole. If more sophisticated methods are used to obtain greater precision of the C–S sinkhole volume, then more accurate implementations of XGBoost can be obtained.

The strain measurements obtained from the FBG strain sensors are promising for using AI to determine when a C–S sinkhole has started growing, is growing, and has stopped growing since the strain patterns vary significantly from phase to phase. Future work will include using more nonparametric AI algorithms and time series analysis to determine which phase the C–S sinkhole is in, investigating more sophisticated techniques for determining the volume of the C–S sinkhole, investigating multicollinearity amongst the predictor variables (i.e., the FBG strain sensors) and investigating data transformations to linearize the relationships between the three FBG strain sensors across all three phases of the C–S sinkhole formation process. In addition, future work will also include investigating how different soil compositions, sensor placements, and sinkhole conditions impact sinkhole volume estimation using FBG strain data.

## Figures and Tables

**Table 1:** Strength of Correlation Guide. Source: [19].

Amount of *ρ*	Strength of Correlation
0.0<0.1	No correlation
0.1<0.3	Low correlation
0.3<0.5	Medium correlation
0.5<0.7	High correlation
0.7<1.0	Very high correlation

**Table 2:** Normal Distribution Test Results for FBG Strain S1 during Phase One.

Empirical Test Method	Statistic	p-Value
K–S Test	0.21	<0.001
S–W Test	0.90	<0.001
A–D Test	28.03	<0.001

**Table 3:** Normal Distribution Test Results for FBG Strain S2 during Phase One.

Empirical Test Method	Statistic	p-Value
K–S Test	0.22	<0.001
S–W Test	0.85	<0.001
A–D Test	46.71	<0.001

**Table 4:** Normal Distribution Test Results for FBG Strain S3 during Phase One.

Empirical Test Method	Statistic	p-Value
K–S Test	0.21	<0.001
S–W Test	0.93	<0.001
A–D Test	22.91	<0.001

**Table 5:** Spearman’s Rank Correlation and Significance Results for FBG Strain S1–S3 during Phase One of the C–S Sinkhole Formation Process.

		FBG Strain S3	FBG Strain S2	FBG Strain S1
**FBG Strain S3**	Spearman’s Rank Correlation	1	0.85	0.87
	p-value	-	<0.001	<0.001
**FBG Strain S2**	Spearman’s Rank Correlation	0.85	1	0.79
	p-value	<0.001	-	<0.001
**FBG Strain S1**	Spearman’s Rank Correlation	0.87	0.79	1
	p-value	<0.001	<0.001	-

**Table 6:** Normal Distribution Test Results for FBG Strain S1 during Phase Two.

Empirical Test Method	Statistic	p-Value
K–S Test	0.38	<0.001
S–W Test	0.53	<0.001
A–D Test	453.07	1

**Table 7:** Normal Distribution Test Results for FBG Strain S2 during Phase Two.

Empirical Test Method	Statistic	p-Value
K–S Test	0.25	<0.001
S–W Test	0.69	<0.001
A–D Test	217.07	<0.001

**Table 8:** Normal Distribution Test Results for FBG Strain S3 during Phase Two.

Empirical Test Method	Statistic	p-Value
K–S Test	0.30	<0.001
S–W Test	0.30	<0.001
A–D Test	∞	

**Table 9:** Normal Distribution Test Results for C–S Sinkhole Volume during Phase Two.

Empirical Test Method	Statistic	p-Value
K–S Test	0.06	<0.001
S–W Test	0.95	<0.001
A–D Test	28.12	<0.001

**Table 10:** Spearman’s Rank Correlation and Significance Results for FBG Strain S1–S3 and the C–S Sinkhole Volume during Phase Two of the C–S Sinkhole Formation Process.

		FBG Strain S3	FBG Strain S2	FBG Strain S1	C–S Sinkhole Volume
**FBG Strain S3**	Spearman’s Rank Correlation	1	0.95	0.40	−0.80
	p-value	-	<0.001	<0.001	<0.001
**FBG Strain S2**	Spearman’s Rank Correlation	0.95	1	0.33	−0.76
	p-value	<0.001	-	<0.001	<0.001
**FBG Strain S1**	Spearman’s Rank Correlation	0.40	0.33	1	−0.48
	p-value	<0.001	<0.001	-	<0.001
**C–S Sinkhole Volume**	Spearman’s Rank Correlation	−0.80	−0.76	−0.48	1
	p-value	<0.001	<0.001	<0.001	-

**Table 11:** Normal Distribution Test Results for FBG Strain S1 during Phase Three.

Empirical Test Method	Statistic	p-Value
K–S Test	0.40	<0.001
S–W Test	0.64	<0.001
A–D Test	∞	

**Table 12:** Normal Distribution Test Results for FBG Strain S2 during Phase Three.

Empirical Test Method	Statistic	p-Value
K–S Test	0.30	<0.001
S–W Test	0.57	<0.001
A–D Test	∞	

**Table 13:** Normal Distribution Test Results for FBG Strain S3 during Phase Three.

Empirical Test Method	Statistic	p-Value
K–S Test	0.38	<0.001
S–W Test	0.55	<0.001
A–D Test	87.74	<0.001

**Table 14:** Spearman’s Rank Correlation and Significance Results for FBG Strain S1–S3 during Phase Three of the C–S Sinkhole Formation Process.

		FBG Strain S3	FBG Strain S2	FBG Strain S1
**FBG Strain S3**	Spearman’s Rank Correlation	1	0.49	0.71
	p-value	-	<0.001	<0.001
**FBG Strain S2**	Spearman’s Rank Correlation	0.49	1	0.49
	p-value	<0.001	-	<0.001
**FBG Strain S1**	Spearman’s Rank Correlation	0.71	0.49	1
	p-value	<0.001	<0.001	-

**Table 15:** WLS Implementation Results for Phase Two Data.

**Training Set Size**	**Regularization**	**Train RMSE**	Test RMSE	$\mathrm{Train}\;R^{2}$	$\mathrm{Test}\;R^{2}$
60%	No penalty	131.80	132.80	0.42	0.43
	L1	131.81	132.71	0.42	0.43
	L2	132.85	133.15	0.42	0.41
70%	No penalty	133.33	129.88	0.41	0.45
	L1	133.34	129.83	0.41	0.45
	L2	134.25	130.89	0.40	0.44
80%	No penalty	133.06	128.39	0.42	0.45
	L1	133.06	128.35	0.42	0.45
	L2	133.96	129.78	0.41	0.44

**Table 16:** SVR Implementation Results for Phase Two Data.

Training Set Size	ϵ	Train RMSE	Test RMSE	$\mathrm{Train}\;R^{2}$	$\mathrm{Test}\;R^{2}$
60%	0.01	97.28	89.42	0.68	0.74
60%	0.1	96.78	89.13	0.69	0.74
60%	1	121.14	119.51	0.51	0.54
70%	0.01	96.10	87.02	0.69	0.75
70%	0.1	94.91	86.45	0.70	0.76
70%	1	122.70	121.96	0.50	0.51
80%	0.01	94.03	87.73	0.71	0.74
80%	0.1	93.38	87.85	0.71	0.74
80%	1	124.70	125.03	0.49	0.48

**Table 17:** XGBoost Implementation Results for Phase Two Data.

Training Set Size	Learning Rate	Train RMSE	Test RMSE	$\mathrm{Train}\;R^{2}$	$\mathrm{Test}\;R^{2}$
60%	0.1	20.20	45.91	0.99	0.93
60%	0.2	13.53	46.35	0.99	0.93
60%	0.3	15.05	46.60	0.99	0.93
70%	0.1	20.97	41.89	0.99	0.94
70%	0.2	12.52	38.37	0.99	0.95
70%	0.3	8.64	40.87	1.00	0.95
80%	0.1	8.01	28.79	1.00	0.97
80%	0.2	9.25	30.58	1.00	0.97
**80%**	0.3	7.20	30.74	1.00	0.97

## Data Availability

The data presented in this study are available upon request from the corresponding author due to ongoing research involving multiple PhD students. Access is restricted to ensure the integrity and confidentiality of the ongoing analyses.
